# The impact of a cognitive impairment support program on patients in an acute care setting: a pre-test post-test intervention study

**DOI:** 10.1186/s12877-023-03930-1

**Published:** 2023-05-01

**Authors:** Amanda Fox, Joel Dulhunty, Emma Ballard, Maria Fraser, Margaret Macandrew, Sally Taranec, Rebecca Waters, Min Yang, Mark Yates, Catherine Yelland, Elizabeth Beattie

**Affiliations:** 1 Centre for Healthcare Transformation, Queensland University of Technology, Brisbane, QLD Australia; 2 School of Nursing, Faculty of Health, Queensland University of Technology, N Block, Victoria Park Road, Kelvin Grove, Brisbane, QLD Australia; 3 Redcliffe Hospital, Anzac Avenue, Redcliffe, Australia; 4 QIMR Berghofer Medical Research Institute, Brisbane, Australia; 5 Ballarat Health Services, Ballarat, Australia

**Keywords:** Acute care, Cognitive impairment, Health Service Research, Older patients, Quality of life, Support program

## Abstract

**Background:**

Patients with cognitive impairment are at greater risk of hospital acquired complications, longer hospital stays, and poor health outcomes compared to patients without cognitive impairment. The Cognitive Impairment Support Program is a multi-disciplinary approach to improve screening rates and awareness of patients with cognitive impairment and guide clinician response and communication during their hospitalisation to improve health outcomes.

**Objective:**

This study evaluated the impact of implementing the Cognitive Impairment Support Program on patient hospital acquired complications, patient reported quality of life and staff satisfaction in an outer metropolitan hospital.

**Design:**

A pre-test post-test design was used to collect data in two 6-month time periods between March 2020 and November 2021.

**Participants:**

Patients aged ≥ 65 years, admitted to a participating ward for > 24 h.

**Intervention:**

The Cognitive Impairment Support Program consisted of four components: cognitive impairment screening, initiation of a Cognitive Impairment Care Plan, use of a Cognitive Impairment Identifier and associated staff education.

**Measures:**

The primary outcome was hospital acquired complications experienced by patients with cognitive impairment identified using clinical coding data. Secondary outcomes were patient quality of life and a staff confidence and perceived organisational support to care for patients with cognitive impairment.

**Results:**

Hospital acquired complication rates did not vary significantly between the two data collection periods for patients experiencing cognitive impairment with a 0.2% (95% confidence interval: -5.7–6.1%) reduction in admissions with at least one hospital acquired complication. Patients in the post intervention period demonstrated statistically significant improvements in many items in two of the Dementia Quality of Life Measure domains: memory and everyday life. The staff survey indicated statistically significant improvement in clinical staff confidence to care for patients with cognitive impairment (*p* = 0.003), satisfaction with organisational support for patients (*p* = 0.004) and job satisfaction (*p* ≤ 0.001).

**Conclusion:**

This study provides evidence that a multicomponent Cognitive Impairment Support Program had a positive impact on staff confidence and satisfaction and patient quality of life. Broader implementation with further evaluation of the multicomponent cognitive impairment intervention across a range of settings using varied patient outcomes is recommended.

## Introduction

Cognitive impairment is defined as a person’s difficulty with memory, cognitive function, communication and reasoning [1]. Around 30% of patients over 70 years of age experience cognitive impairment during hospitalisation [2]. A cognitive impairment in adults may include mild cognitive impairment, dementia, delirium or a combined acute on chronic presentation [3]. Dementia is a collection of symptoms such as reduced ability to think, remember and complete everyday tasks caused by disorders that affect the brain [4]. The number of people living with dementia is expected to double by 2058 [4], placing significant challenges on the health care system.

In the acute hospital environment, experiencing cognitive impairment places an older patient at higher risk of experiencing adverse outcomes with 28% of patients admitted to hospital with dementia experiencing an adverse event [5]. Patients with cognitive impairment are at increased risk of falls [6, 7], greater functional decline [8, 9], prolonged length of hospital stay [10], hospital acquired complications such as urinary tract infection, pressure ulcers, pneumonia and delirium [7, 11], unplanned entry to residential care [12] and increased post discharge mortality [13]. These adverse events are at considerable cost to individual and carer health and wellbeing and to the health care system.

In 2018, the Australian Commission on Safety and Quality in Healthcare introduced a national Comprehensive Care Standard, which requires health services to improve screening of patients to ensure early identification of cognitive impairment and implement processes to mitigate risks associated with hospitalisation [14]. Healthcare is hindered when staff have inadequate understanding of the importance of screening and skills for the detection of cognitive impairment [15, 16]. Effective cognitive impairment screening using a validated tool, combined with education for the healthcare workforce that focuses on understanding, supporting and managing patient symptoms and changed behaviours, have been recommended to improve patient outcomes [17–19]. Programs adopting multi-faceted strategies to address risk factors, improve recognition and enhance management of patients with cognitive impairment have been associated with positive work environments and improved care for older patients [20, 21]. The Dementia Care in Hospitals Program (DCHP) is one such program, with the addition of an over bedside alert, the Cognitive Impairment Identifier (CII) to assist staff awareness. The CII was developed in collaboration with consumers, clinicians and researchers and has been endorsed by Dementia Australia to alert clinicians to a patient’s cognitive impairment [22]. The program was introduced at Ballarat Health Services in 2004 with a focus on raising detection rates of cognitive impairment and has since been implemented in a number of hospitals across four Australian states [23, 24]. Evaluation of the program showed a 14% reduction in hospital acquired complications for all patients, significant improvement of staff confidence in caring for patients with cognitive impairment, increased staff job satisfaction and organisational support, and greater self-reported carer satisfaction with the care provided to their relatives [23, 25]. Against this background, the aim of this study was to evaluate the impact of implementing a Cognitive Impairment Support (CIS) Program, which was based on the DCHP but adapted to the local context and used contemporary evidence based patient interventions, on hospital acquired complication (HAC) rates for patients in an acute care setting, patient quality of life and staff confidence.

## Methods

### Study design and setting

**Fig. 1:**
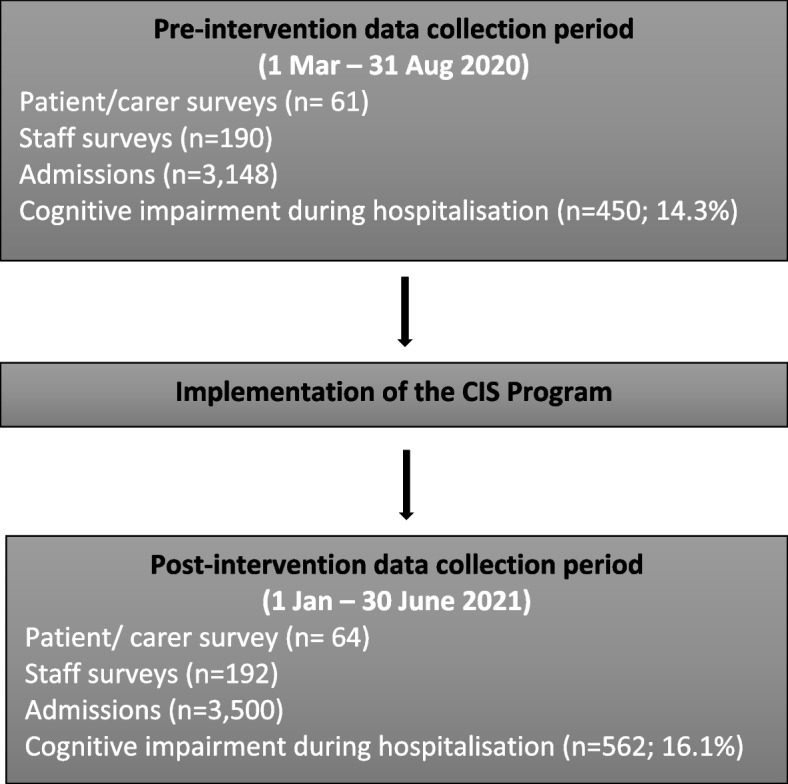
Study Flow chart

This study was conducted in a 250-bed outer-metropolitan public teaching hospital with approximately 15,000 medical and surgical admissions per year. A pre-test post-test study design was used to evaluate the impact of the CIS Program implemented across five acute hospital wards (two medical wards, a general surgical ward, an orthopaedic ward and a short stay ward) comprising approximately 72% of all hospital admissions. Paediatric, obstetric, intensive care and coronary care wards were excluded. There were approximately 300 nursing, 135 medical and 30 allied health staff employed in the study wards. Study timelines are shown in Fig. 1. Patients in the pre-intervention group were discharged between 1 March 2020 and 31 August 2020. Patients in the post-intervention group were discharged between 1 January 2021 and 30 June 2021. This study is reported in accordance with the Strengthening the Reporting of Observational Studies in Epidemiology (STROBE) guidelines [26]. Ethics approval was obtained from The Prince Charles Hospital Human Research Ethics Committee.

### Participants

There were two participant groups in this study: patients aged ≥ 65 years (or ≥ 45 years for Aboriginal and Torres Strait Islander people) admitted to one or more of the participating wards for longer than 24 h during the data collection periods, and healthcare staff who had direct contact with patients experiencing cognitive impairment in the participating wards. International Statistical Classification of Disease (ICD-10) codes were used to identify patients diagnosed with cognitive impairment during their stay. Staff participants included nurses, doctors and allied health, administration and operational staff, including wards persons, cleaners, security personnel and food services staff.

### Intervention

The CIS Program was based on the DCHP with modification to suit the local context [23]. The modification process involved use of contemporary literature, collaboration and agreement between the investigative team, multidisciplinary clinicians and consumers on modifications to an existing Cognitive Impairment Care Plan and use of the screening tool. The CIS Program comprised four components: (1) increasing the rate of cognitive impairment screening of all patients 65 years of age, or > 45 years of age for Aboriginal or Torres Strait Islander people, using the 4AT tool, (2) embedding use of a Cognitive Impairment Care Plan that prompted initiatives such as the Top 5 (Talk to the carer, Obtain the information, Personalise the care and 5 strategies developed) [27], (3) use of the Cognitive Impairment Identifier (CII) at the patient’s bedside [22] and (4) an educational program for clinical and non-clinical hospital staff to raise awareness of the needs of patients with cognitive impairment and the most suitable communication techniques. The 4AT tool [28] was used by clinical staff to screen and detect patients experiencing cognitive impairment and inform the care provided to the patient. The 4AT was conducted with patients on admission, daily and if a clinician was concerned about a change in patient cognition. The tool contains four screening questions, scored from 0–12 and has been shown to be clinically valid for cognitive impairment screening in older adult patients [19, 29]. A threshold of ≥ 4 on the 4AT tool, or a diagnosis of dementia or delirium, indicated the use of the Cognitive Impairment Care Pathway and the CII at the patient’s bedside. While the intervention was not specifically aimed to reduce HACs, it did focus on identification of patients experiencing cognitive impairment and education of clinicians to respond appropriately to these patient needs. HACs were used as a patient outcome following implementation to measure the effectiveness of the program as has been reported in previous work [23].

### Measures

The predefined primary outcome measure was the number of admissions where patients with cognitive impairment had at least one HAC. A HAC was defined as a secondary diagnosis recorded on discharge of either pneumonia, urinary tract infection, pressure injury, fall or onset of cognitive impairment during the hospital admission. These HACs were selected as being potentially modifiable through care provided to patients by healthcare staff [7]. Patient HACs and cognitive impairment diagnoses were identified using ICD-10 codes extracted from the hospital clinical and administrative database.

Predefined secondary outcome measures included patient reported quality of life and staff confidence and satisfaction with organisational support. Patient quality of life were assessed using the Dementia Quality of Life (DEMQoL) survey, a 28-item interviewer-administered questionnaire asking participants to consider their experiences over the past week [30]. The survey is divided into three domains: Feelings (13 items), Memory (6 items) and Everyday life (9 items). Individual items are scored 1 (a lot), 2 (quite a bit), 3 (a little) and 4 (not at all), with a higher score indicating better health-related quality of life. Data were collected from a sample of patients with cognitive impairment admitted to the participating wards during the data collection periods, or their care partners, at discharge or at day five of hospitalisation, whichever was sooner. The staff survey included eight items, three demographic questions and five Likert scale questions as used in the evaluation of the program [23]. Response options were 1 (very low), 2 (low), 3 (satisfactory), 4 (high) and 5 (very high). A sample of staff, who were employed in the participating wards during the data collection periods were surveyed during each data collection period.

### Sample size

A sample size of 564 admissions of patients with cognitive impairment in each time period was determined to have sufficient power (80%) to detect a clinically significant reduction of 8% in a pre-intervention HAC event rate of 40% using a chi-squared test with alpha of 5%. After accounting for a drop-out rate of up to 6%, the target sample size for each period was 600 patients with cognitive impairment. The target sample size for the DEMQoL patient survey (*n* = 100) and staff survey (*n* = 200) was determined based on how many participants were likely to respond based on previous survey response rates.

### Statistical analysis

The primary outcome was reported as the reduction in admissions with at least one HAC reported as an odds ratio with 95% confidence interval. Additional exploratory analysis was undertaken to check the effect of patients with repeated admissions on the odds ratio using a mixed effects logistic regression model with group as a fixed effect and subject as a random effect.

Patient DEMQoL survey responses were categorised into binary variables of yes (responses 1 or 2) and no (responses 3 or 4) post data collection based on the responses. This was applied to all survey items to improve interpretability. Staff survey responses were also categorised post collection into binary variable of a negative rating (responses 1 or 2) to a positive rating (responses 3, 4 or 5) and results stratified by clinical and non-clinical staff. Categorical variables were summarised as frequency and per cent and continuous variables as mean and standard deviation (SD) or median and inter-quartile range (IRQ) if not normally distributed. Categorical variables were examined using a Pearson Chi-squared test or Fisher’s Exact test. Continuous variables were examined using a Student’s t-test or Mann–Whitney U test. A two-sided *p* value < 0.05 was considered statistically significant with no adjustment made for multiple comparisons. Data were analysed using SPSS versions 27 and 28 (IBM Corp., Armonk, NY).

## Results

### Patient characteristics

There was a total of 3,148 admissions (2447 patients) in the pre-intervention period and 3,500 admissions (2732 patients) in the post-intervention period. In the pre-intervention group, 14.3% of admissions (450/3148) had a cognitive impairment diagnoses either on or during their hospital admission compared with 16.1% (562/3500) in the post-intervention group (*p* = 0.046). Sixty-one and 64 patient/carer participants completed the DEMQoL measure in the pre-intervention and post-intervention periods, respectively. Staff confidence and satisfaction surveys were completed by 190 and 192 staff in the pre-intervention and post-intervention periods respectively. The study flow chart is provided in Fig. 1. Patient characteristics for all admissions are described in Table 1. Table 2 describes characteristics for patient admissions experiencing cognitive impairment. Patient characteristics where similar across the two time periods for all general patient admissions and those patients with cognitive impairment.

**Table 1:** Patient characteristics for all admissions during the study periods

Characteristic	Pre-intervention	Post-intervention	*p*-value
	(*n* = 3148)	(*n* = 3500)	
	n (%)	n (%)	
Gender^a^ (male, *n* = 5179)	1202 (49.1)	1357 (49.7)	0.69
Indigenous status^a^ (*n* = 5175)	99 (4.0)	110 (4.0)	0.98
Age at admission (years, mean (SD))	78.2 (9.4)	78.2 (9.0)	0.91
Admission ward			
Medical	2176 (69.1)	2403 (68.7)	
Surgical	972 (30.9)	1097 (31.3)	0.68
CI recorded in chart on admission	173 (5.5)	222 (6.3)	0.15
Length of stay (days, median (IQR))	3 (2—6)	3 (2—6)	0.90
In-hospital mortality	23 (0.7)	45 (1.3)	0.025

^a^ number of patients vs admissions being presented for the other variables

**Table 2:** Characteristics of patients with cognitive impairment during the study periods

Characteristic	Pre-intervention	Post-intervention	*p*-value
	(*n* = 450)	(*n* = 562)	
	n (%)	n (%)	
Gender^a^ (male, *n* = 802)	167 (46.5)	225 (50.8)	0.23
Indigenous status^a^ (*n* = 801)	6 (1.7)	8 (1.8)	0.82
Age at admission (years, mean (SD))	82.5 (8.3)	82.9 (8.2)	0.46
Admission ward			
Medical	385 (85.6)	495 (88.1)	
Surgical	65 (14.4)	67 (11.9)	0.24
Length of stay (days, median (IQR))	5 (3 – 10)	5 (3 – 9)	0.10
In-hospital mortality	9 (2.0)	8 (1.4)	0.48

^a^ number of patients vs admissions being presented for the other variables

### Hospital-acquired complications

There was no statistically significant difference between time periods in the type of HAC or the number of admissions with at least one HAC (Table 3). There was a 0.2% (95% CI -5.7—6.1) reduction in admissions with at least one HAC following the intervention. The unadjusted odds of having at least one HAC following the intervention for patients with cognitive impairment was 0.99 (95% CI 0.76—1.28) and the adjusted odds was 0.98 (95% CI 0.72—1.31) compared to patients with cognitive impairment in the pre-intervention period.

**Table 3:** Hospital acquired complications experienced by patients with cognitive impairment during the study periods

Hospital acquired complications	Pre-intervention	Post-intervention	*p*-value
	(*n* = 450)	(*n* = 562)	
	n (%)	n (%)	
Type of HAC			
CI onset in hospital	277 (61.6)	340 (60.5)	0.73
Urinary tract infection	9 (2.0)	5 (0.9)	0.13
Pressure injury	14 (3.1)	12 (2.1)	0.33
Pneumonia	14 (3.1)	17 (3.0)	0.94
Fall	28 (6.2)	43 (7.7)	0.38
Number of HACs (*n* = 651, median (IQR))	1 (1–1)	1 (1–1)	0.48
At least one HAC	290 (64.4)	361 (64.2)	0.95

### Patient quality of life measure

Patients surveyed during the pre and post intervention periods did not vary in their perception of their overall health, however results showed statistically significant improvements across many of the individual DEMQoL items in two of the three domains: Memory and Everyday life (Table 4).

**Table 4:** Number of patients who responded ‘yes’ to individual quality of life measures during the study periods

Domain / item	Pre-intervention	Post-intervention	*p*-value
	*n* = 61	*n* = 64	
	n (%)	n (%)	
**Feelings**			
Cheerful^a^	32 (52.5)	36 (56.3)	0.67
Worried or anxious	28 (45.9)	19 (29.7)	0.06
Enjoying life^a^	26 (43.3)	23 (35.9)	0.40
Frustrated	25 (41.0)	27 (42.2)	0.89
Confident^a^	23 (39.0)	16 (25.0)	0.10
Full of energy^a^	18 (30.0)	10 (15.6)	0.056
Sad	24 (39.3)	17 (26.6)	0.13
Lonely	19 (31.1)	16 (25.0)	0.44
Distressed	23 (37.7)	14 (21.9)	0.053
Lively^a^	17 (28.8)	7 (10.9)	0.012
Irritable	20 (32.8)	13 (20.3)	0.11
Fed-up	30 (49.2)	21 (32.8)	0.063
Things you wanted to do but couldn't	42 (71.2)	31 (48.4)	0.010
**Memory**			
Forgetting things	29 (47.5)	12 (18.8)	< 0.001
Forgetting people	19 (31.1)	7 (10.9)	0.005
Forgetting day	23 (37.7)	12 (18.8)	0.018
Thoughts muddled	25 (41.0)	15 (23.8)	0.041
Difficulty making decisions	20 (32.8)	8 (12.5)	0.007
Poor concentration	18 (30.5)	11 (17.2)	0.082
**Everyday life**			
Not having enough company	16 (26.2)	12 (18.8)	0.32
Getting on with people close to you	13 (21.3)	2 (3.2)	0.002
Getting affection	15 (24.6)	5 (7.8)	0.011
People not listening	25 (41.0)	11 (17.5)	0.004
Making yourself understood	17 (28.3)	8 (12.5)	0.028
Getting help when needed	18 (29.5)	12 (18.8)	0.16
Getting to the toilet in time	17 (27.9)	15 (23.4)	0.57
How you feel	17 (27.9)	10 (15.6)	0.096
Your health overall	18 (29.5)	19 (29.7)	0.98

^a^ Items were reversed before calculating the total score

### Staff confidence

The characteristics of staff who completed the pre and post intervention surveys are presented in Table 5. The participants consisted of similar proportions of healthcare professionals and operational staff during both data collection periods.

**Table 5:** Professional group of staff completing surveys during the study periods

Staff position	Pre-intervention	Post-intervention	*p*-value
	(*n* = 190)	(*n* = 192)	
	n (%)	n (%)	
Administration	25 (13.2)	21 (10.9)	0.56
Allied health	23 (12.1)	30 (15.6)	0.34
Medical	22 (11.6)	22 (11.4)	1.00
Nursing	89 (46.8)	89 (46.4)	1.00
Operational	31 (16.3)	30 (15.6)	0.90

Staff surveys conducted pre and post intervention indicate significant improvement across many of the survey items (Table 6). In particular, clinical staff appear to have gained significant confidence, comfort, perception of organisational support and job satisfaction caring for patients with a cognitive impairment. They were also more satisfied after the CIS program that the hospital is well equipped to care for patients with cognitive impairment.

**Table 6:** Number of staff who reponded ‘yes’ to the survey during the study periods

Item	Pre-intervention	Post-intervention	*p*-value
	Clinical *n* = 134	Clinical *n* = 141	
	Non- clinical *n* = 56	Non- clinical *n* = 51	
	n (%)	n (%)	
**Level of confidence**			
*Clinical staff*	103 (78.6)	129 (91.5)	0.003
*Non-clinical staff*	44 (78.6)	38 (76)	0.75
**Level of comfort**			
*Clinical staff*	107 (81.7)	127 (90.7)	0.030
*Non-clinical staff*	46 (82.1)	35 (71.4)	0.19
**Level of organisational support**			
*Clinical staff*	87 (64.9)	112 (80.6)	0.004
*Non-clinical staff*	37 (67.3)	35 (68.6)	0.88
**Job satisfaction**			
*Clinical staff*	78 (58.6)	114 (81.4)	< 0.001
*Non-clinical staff*	41 (73.2)	40 (80.0)	0.41
**Well-equipped hospital environment**			
*Clinical staff*	63 (47.4)	86 (61.9)	0.016
*Non-clinical staff*	35 (67.3)	31 (62.0)	0.58

^a^Not all participants answered all items in the survey

## Discussion

The aim of this study was to evaluate the impact of implementing the CIS Program on HAC rates for patients experiencing cognitive impairment in an acute care setting. The number of admissions with at least one HAC in patients with cognitive impairment in the post-intervention period was slightly higher than in the pre-intervention period. The reported rate of cognitive impairment (14.3% and 16.1% during hospitalisation pre and post intervention respectively) is significantly lower than the previously published 30% of patients aged ≥ 70 years [31], however this study included all adult hospital admissions limiting direct comparison.

The primary outcome measure was the number of admissions where patients with cognitive impairment experienced at least one HAC (cognitive impairment onset in hospital, urinary tract infections, pressure injuries falls and pneumonia) which occurred in 64.4% of admissions pre intervention and 62.4% post intervention and the dominance of cognitive impairment onset in hospitals. These values are reliant on appropriate reporting of cognitive impairment and where the onset occurred. Interestingly, the number of patients with cognitive impairment reported to have experienced a fall increased slightly post-intervention. Many factors may have influenced this result, however raising staff awareness and improved identification may have led to increased reporting of in-hospital falls.

Implementation of the CIS Program did indicate a statistically significant improvement in aspects of patient quality of life measured in two out of three domains. This finding contrasts to the DCHP study, which did not find any significant difference in quality of life in patients with dementia post-implementation of the program [23]. We found that patients or care partners reported that patient’s memory and everyday life domains were significantly improved post implementation of the CIS Program. This may have been due to early detection and appropriate use of the Cognitive Impairment Care Plan that actioned use of resources to remind patients of information such as the date, month, and other simple re-orienting measures. Regularly re-orientating cognitively impaired patients to the time, date and place may improve cognitive function [32]. In addition, the Cognitive Impairment Care Plan prompted clinicians to individualise patient care in a meaningful way, along with initiating appropriate use of the CII above the patient’s bed [22]. This identifier prompts staff who attended an educational session to use appropriate and simplified communication strategies that allowed additional time for patients to answer questions and make decisions which may be reflected in the improved quality of life scores for everyday life.

All hospital staff working with patients who experience cognitive impairment were included in this study. This ‘whole of hospital’ approach was employed due to the frequent exposure that clinical and operational staff have to patients with cognitive impairment and the opportunity they have to improve the hospital experience and outcomes for patients. There were statistically significant improvements across all five survey items for allied health, medical and nursing (clinical) staff following implementation of the CIS Program, consistent with research by Murray and colleagues [25] who evaluated staff satisfaction following implementation of the DCHP in four Australian hospitals. Research further suggests that education of staff can influence confidence when managing behaviours associated with cognitive impairment [33] and clinician career development, professional values and high job satisfaction are positively correlated with retention in the profession [34]. Retaining experienced clinicians in healthcare roles is imperative to providing quality care. Job satisfaction and staff retention of staff has been linked to teamwork and nursing staff perceptions of patient safety [35].

Conversely, operational staff did not report the same level of satisfaction with caring for patients with cognitive impairment. Operational staff (e.g. wards persons, cleaners, catering and administration staff) reported lower rates of confidence and comfort compared to their clinician counterparts. After the CIS Program which included an educational session aimed at improving the awareness and communication strategies for people with cognitive impairment, operational staff may have had increased awareness of what they did not know, also known as the Dunning-Kruger confidence vs. knowledge cognitive bias [36]. This may have led to a decreased confidence and comfort in caring for patients experiencing cognitive impairment being reported. This discrepancy between clinical and non-clinical effectiveness of the CIS Program may indicate a greater need for educational support for these staff. There may have also been discipline specific culture, leaders’ attitudes, varied dissemination and delivery of education that occurred due to the impact of COVID-19.

### Limitations

This study was undertaken at one outer metropolitan hospital, which may limit generalisability to other settings. This research project experienced some implementation and data collection delay associated with the SARS-CoV-2 (COVID-19) pandemic. Implementation of the CIS Program occurred in a mix of face-to-face and online interactions with staff, rather than all face-to-face education as initially planned, and care partner surveys were unable to be undertaken due to visitation restrictions during the COVID-19 pandemic. Despite this, the impact of COVID-19 was similar across both data collection periods in relation to visitor and service provision restrictions. The distribution of patient admissions across each month per year during the study period was examined with no obvious differences between years observed. While target sample sizes were not fully achieved (particularly in the pre-intervention period), the size of the drop in HAC post intervention was a quarter of the expected 8% and it is unlikely that this value would have achieved statistical significance with a fully powered study. As no adjustment was made for multiple comparisons there is an increased chance of a type 1 error and the size of the difference between pre and post intervention periods should be taken into consideration when interpreting results.

This non-randomised intervention study was conducted in a complex health care setting over an extensive implementation period, so may have been impacted by internal validity threats and extraneous factors, for example, seasonality of data collecting periods, clinical coding limitations and other unaccounted for biases.

## Conclusion

This study provides evidence that a multicomponent CIS Program targeting screening for cognitive impairment, staff education and use of a CII as part of a Cognitive Impairment Care Plan had the greatest impact on staff confidence and satisfaction and patient quality of life. Direct impact on HACs was not observed, however, longer term follow-up may be required to observe downstream impacts of the CIS Program. Further implementation with evaluation of a multicomponent cognitive impairment intervention across varied settings to evaluate other patient outcome variables such as functional independence measures and with greater focus on non-clinical staff is recommended based on this experience of implementing the CIS Program.

## Data Availability

The datasets used and/or analysed during the current study are available from the corresponding author on reasonable request (a.fox@qut.edu.au).
